# Revealing the contribution of astrocytes to glutamatergic neuronal transmission

**DOI:** 10.3389/fncel.2022.1037641

**Published:** 2023-01-19

**Authors:** Ares Orlando Cuellar-Santoyo, Victor Manuel Ruiz-Rodríguez, Teresa Belem Mares-Barbosa, Araceli Patrón-Soberano, Andrew G. Howe, Diana Patricia Portales-Pérez, Amaya Miquelajáuregui Graf, Ana María Estrada-Sánchez

**Affiliations:** ^1^División de Biología Molecular, Laboratorio de Neurobiología, Instituto Potosino de Investigación Científica y Tecnológica (IPICYT), San Luis Potosí, Mexico; ^2^Translational and Molecular Medicine Laboratory, Research Center for Health Sciences and Biomedicine, Autonomous University of San Luis Potosí, San Luis Potosí, Mexico; ^3^Intelligent Systems Laboratory, HRL Laboratories, LLC, Malibu, CA, United States; ^4^Faculty of Medicine and Health Sciences, University of Antwerp, Antwerp, Belgium

**Keywords:** calcium, gliotransmission, NMDA receptors, GLT-1, GLAST, VGLUT1, xCT

## Abstract

Research on glutamatergic neurotransmission has focused mainly on the function of presynaptic and postsynaptic neurons, leaving astrocytes with a secondary role only to ensure successful neurotransmission. However, recent evidence indicates that astrocytes contribute actively and even regulate neuronal transmission at different levels. This review establishes a framework by comparing glutamatergic components between neurons and astrocytes to examine how astrocytes modulate or otherwise influence neuronal transmission. We have included the most recent findings about the role of astrocytes in neurotransmission, allowing us to understand the complex network of neuron-astrocyte interactions. However, despite the knowledge of synaptic modulation by astrocytes, their contribution to specific physiological and pathological conditions remains to be elucidated. A full understanding of the astrocyte’s role in neuronal processing could open fruitful new frontiers in the development of therapeutic applications.

## Introduction

The brain is one of the most complex organs in the human body; it consists of many distinct cell types, but most cells fall under the broad categories of neurons or glia with approximately even numbers in each category. The glial cells are divided into microglia, oligodendrocytes, and astrocytes; the latter comprise around 20% of the cells in the brain (Ventura and Harris, 1999; Salas et al., 2020). Astrocytes participate in many neurophysiological processes, including synaptogenesis (Allen and Eroglu, 2017), modulation of synaptic transmission, neuronal plasticity (Newman and Zahs, 1998; Araque et al., 1999), and regulation of blood flow in addition to the trafficking of small molecules and ions through their end-feet processes at the blood-brain barrier (Giaume et al., 1997; Simard et al., 2003). During physiological conditions, neurons and astrocytes have a coordinated functional relationship that ensures proper information flow, and each contributes to synaptic transmission by releasing neurotransmitters (by presynaptic neurons) or gliotransmitters (by astrocytes; see below).

Glutamate is the primary excitatory neurotransmitter in the mammalian brain, and it participates in diverse physiological processes such as learning, memory, and neuronal development (Yu et al., 1984; Behar et al., 1999; Hrabetova et al., 2000). However, glutamate can induce neuronal damage through excitotoxicity, which results from the over-activation of glutamatergic receptors. Glutamate-mediated toxicity has been implicated in the pathogenesis of neurodegenerative diseases including Alzheimer’s, Huntington’s, and Parkinson’s diseases (Koutsilieri and Riederer, 2007; Estrada-Sánchez et al., 2009; Ong et al., 2013), as well as psychiatric disorders such as schizophrenia (O’Donovan et al., 2017; Shah et al., 2020). Although glutamate toxicity contributes to these neuropathological conditions, the changes that lead to impaired glutamatergic neurotransmission are diverse, with different causes in each pathology. Nonetheless, an impaired relationship between neurons and astrocytes might be a common component. In the next section, we review the similarities and differences among the components of glutamatergic transmission in neurons and astrocytes.

## Glutamatergic neurotransmission

### Glutamate synthesis

**Figure 1:**
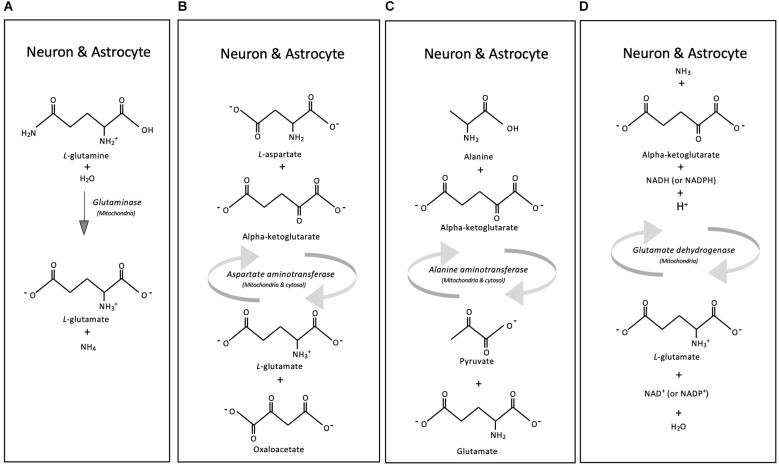
Glutamate synthesis. Schematic representation of four main metabolic reactions, synthesizing glutamate in neurons and astrocytes: Glutaminase **(A)**, Aspartate aminotransferase **(B)**, Alanine aminotransferase **(C)**, and Glutamate dehydrogenase **(D)**.

Glutamate plays a role in multiple biological processes in the brain, and yet it cannot cross the blood-brain barrier. Instead, the brain’s glutamate is synthesized locally by astrocytes and neurons through one of several pathways. We provide a brief description of glutamate metabolism (this section and Figure 1; Hawkins, 2009; Fernstrom, 2018); further details can be found in the extensive review by Schousboe et al. (2014).

The main precursor of glutamate in the brain is glutamine, a key component of the glutamine-glutamate cycle that encompasses the exchange of glutamine and glutamate between astrocytes and neurons. The reuptake of glutamate by its transporters, glutamate transporter 1 (GLT-1) and glutamate aspartate transporter (GLAST) in astrocytes enables the synthesis of glutamine by the cytosolic enzyme glutamine synthetase, which is highly expressed in astrocytes. Glutamine synthetase promotes the conversion of glutamate to glutamine using the cofactors NADPH, ATP, and NH^+^_4_ (Lehre et al., 1995; Bergles and Jahr, 1997; Anlauf and Derouiche, 2013; Huyghe et al., 2014; Yamada et al., 2019). Glutamine is then transported to the synaptic cleft, where neurons take it up through specific glutamine transporters (i.e., members of the families SNAT, LAT, ASC, and ^+^LAT; Anlauf and Derouiche, 2013; Yamada et al., 2019). Once transported into neurons, the mitochondrial enzyme glutaminase (an amidohydrolase) generates glutamate from the glutamine provided by the astrocytes (Figure 1A). Glutamate synthesis occurs predominantly in neurons, although astrocytes can synthesize it through the same pathway (Hogstad et al., 1988). Neurons and astrocytes both preferentially express the mitochondrially-located, kidney-type glutaminase (GLS) isoenzyme type 1 (GLS1), although there is also the liver-type GLS2 that localizes to the mitochondrion and nucleus (Cardona et al., 2015). However, evidence suggests that neurons and astrocytes might express an isoform of glutaminase GLS1, which has not been characterized to date (Kvamme et al., 2001; Cardona et al., 2015).

Glutamate is a component of energy metabolism, which requires *de novo* synthesis to avoid an imbalance in glutamate concentrations. *De novo* glutamate synthesis occurs by the pyruvate carboxylase, which is located exclusively in astrocytic mitochondria; this enzyme metabolizes pyruvate into oxaloacetate, a precursor for α-ketoglutarate (Walker, 2014; Schousboe et al., 2019). Although neurons lack pyruvate carboxylase, they contribute to *de novo* glutamate synthesis by the pyruvate carboxylation to malate through the malic enzyme, which is found in the cytosol and mitochondria (McKenna et al., 1995; Hassel, 2001; Amaral et al., 2016).

Another enzyme that contributes to glutamate production is aspartate aminotransferase (found in the cytosol or mitochondria), which synthesizes glutamate by reversibly transferring the α-amino group from aspartate to 2-oxoglutarate, resulting in glutamate and oxaloacetate; this enzyme uses pyridoxal 5’-phosphate as a co-factor (Figure 1B; McKenna et al., 2006; Schousboe, 2017). Neurons and astrocytes express aspartate aminotransferase, and the enzyme appears to have the same function and activity in both cell types (McKenna et al., 2006).

Astrocytes and neurons contain alanine aminotransferase in the cytoplasm and mitochondria (Ruscak et al., 1982; Waagepetersen et al., 2000), which catalyzes the reversible interconversion of alanine and α-ketoglutarate into pyruvate and glutamate (Figure 1C). Low activity of this enzyme in neurons (Westergaard et al., 1993; Erecinska et al., 1994) suggests that, within this pathway, astrocytes exert a primary control (Schousboe et al., 2013).

Ammonia concentration in the brain is regulated by the mitochondrial glutamate dehydrogenase, which catalyzes the reversible conversion of glutamate to α-ketoglutarate and ammonia, using NADH or NADPH as a co-factor (Islam et al., 2010; Plaitakis et al., 2017). Glutamate dehydrogenase expression in astrocytes varies spatially by brain region, cellularly by astrocyte type, and temporally with the developmental stage (Figure 1D; Osterberg and Wattenberg, 1962). For example, astrocytes increase glutamate dehydrogenase expression during rat hippocampus maturation (Kugler and Schleyer, 2004). Interestingly, along with increased glutamate dehydrogenase activity, astrocytes also increase the expression of GLT-1, suggesting a deeper, interconnected regulatory system of glutamatergic dynamics (Kugler and Schleyer, 2004).

### Glutamate packaging

In neurons, glutamate is packaged and stored in synaptic vesicles through specific vesicular glutamate transporters (VGLUT). Currently, three subtypes of VGLUTs (VGLUT1, 2, and 3) have been described, and their distribution differs among different brain structures. VGLUT1 is present in the cerebral cortex, cerebellum, hippocampus, and thalamus (Fujiyama et al., 2001; Herzog et al., 2004). VGLUT2 is expressed in the cortex, thalamus, diencephalon, and rhombencephalon (Fremeau et al., 2001; Herzog et al., 2004). VGLUT3 is less predominant than the other two transporters and is located in the striatum, neocortex, and hippocampus (Fremeau et al., 2002).

VGLUT function depends on the electrochemical proton gradient generated across the membrane by the activity of the vacuolar H^+^-ATPase (Wolosker et al., 1996). This H^+^-ATPase activity increases the H^+^ concentration inside the vesicle leading to an acidic pH. The rate of glutamate transport by VGLUT correlates inversely with the concentration of chloride ions (Cl^−^) such that a low extravesicular Cl^−^ concentration generates high glutamate uptake, whereas high Cl^−^ concentration leads to gradual inhibition of glutamate uptake (Wolosker et al., 1996; Juge et al., 2006).

There appears to be a proportional relationship between VGLUT levels and glutamatergic synapse response. A study of VGLUT1-knockout mice demonstrated that the knockout reduced the amplitude of miniature excitatory postsynaptic currents (mEPSCs), suggesting a smaller quantal size (Wojcik et al., 2004). In another study, vesicles containing a lower number of VGLUT1 showed a reduced release probability (Herman et al., 2014). Similar results have been described also in VGLUT3-knockout mice (Fasano et al., 2017).

The evidence for astrocyte expression of VGLUT is contradictory and requires further investigation to clarify the situation (for a review see Hamilton and Attwell, 2010). For example, Li et al. (2013a) describe the absence of VGLUT in mouse cortical, hippocampal, and cerebellar astrocytes. On the contrary, Ormel et al. (2012) identified VGLUT1 in astrocytic processes in the rat hippocampus, frontal cortex, and striatum. Likewise, astrocytes from postnatal rat brains express VGLUT1 and VGLUT2 (Montana et al., 2004), as well as cortical cultured astrocytes (Anlauf and Derouiche, 2005). VGLUT3 has been detected in astrocytes end-feet in microcultures of rat ventral tegmental area, substantia nigra pars compacta, and raphe nuclei (Fremeau et al., 2002). Despite the controversy about astrocytic VGLUTs, evidence indicates that astrocytes contain vesicular compartments and the molecular machinery to release glutamate in vesicular packages; this phenomenon—now known as gliotransmission—was later confirmed and contributes to neuronal information processing (see below; Bezzi et al., 2004).

### Glutamate release

**Figure 2:**
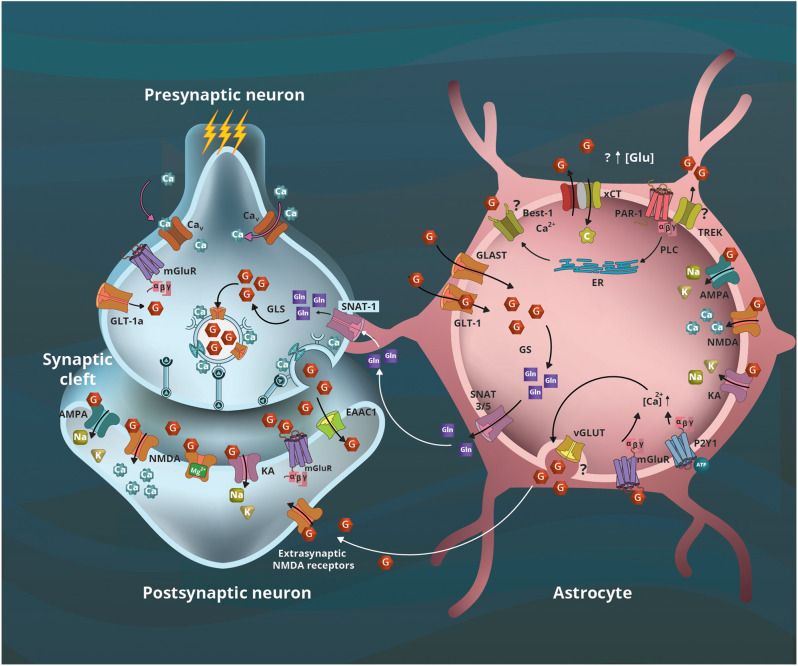
Astrocyte’s contribution to glutamatergic neurotransmission. The arrival of action potential and calcium (Ca^2+^) influx leads to presynaptic vesicular glutamate (G) release to the synaptic cleft, and the activation of its ionotropic (AMPA, NMDA, KA) and metabotropic (mGluR) receptors located in the postsynaptic terminal and astrocytes. The remaining neurotransmitter is captured by transporters located in neurons (EAAC and GLT-1a) and astrocytes (GLT-1 and GLAST). In the former, G is metabolized to glutamine (Gln) by the glutamine synthetase (GS), the initial step of the glutamate-glutamine cycle between astrocytes and neurons that use Gln as a precursor for G. Sodium Neutral Amino acid Transporter (SNAT) 3/5 transporters located in astrocytes will transport Gln to the synaptic space where the neuronal SNAT-1 transporter will internalize it to serve as a precursor for G by glutaminase (GLS) activity. The xCT transporters (SLC7A11) internalize cystine (C) cotransporting G outside the astrocytes; this is the first step of glutathione synthesis that involves astrocytes and neurons. Furthermore, a mechanism involving the interaction of protease-activated receptor 1 (PAR-1) with the potassium channel TREK-1 or Best-1, and the G transported by xCT could contribute to increased extracellular G concentrations. In astrocytes, the activation of mGLuR and purinergic P2Y1 receptors contribute to increased intracellular Ca^2+^ concentration, leading to vesicular G release that activates extrasynaptic NMDA receptors; however, the vesicular proteins involved remain controversial. Modified from Estrada-Sánchez and Rebec (2012).

Once glutamate is packaged into the synaptic vesicles and stored in the synaptic bouton, it is ready to be released upon the arrival of an action potential, which will induce the opening of voltage-dependent calcium channels, increasing intracellular calcium (Ca^2+^) concentration. The Ca^2+^ influx facilitates vesicle fusion with the plasma membrane, which releases the neurotransmitters into the synaptic cleft (Figure 2; de Wit et al., 2009). The soluble N-ethylmaleimide-sensitive factor attachment protein receptor (SNARE) family of proteins are key components for this process. These proteins are divided into two groups, the vesicular v-SNARE, highly abundant in the vesicles, and the target t-SNARE, highly expressed in the target zone in the cellular membrane (Han et al., 2017). A trans-SNARE complex -interaction between v and t SNAREs– must be formed to release the neurotransmitter; the main v-SNAREs are synaptobrevin, synaptotagmin, syntaxin, and the main t-SNARE is SNAP-25.

SNARE expression is not limited to neurons; SNAREs support gliotransmitter release in astrocytes (Crippa et al., 2006). Cultured astrocytes express synaptobrevin II and release glutamate that is reduced by the inhibitors of the neuronal exocytosis botulinum toxin-A and botulinum toxin-C, suggesting the expression of SNAP-25 and syntaxin in astrocytes (Jeftinija et al., 1997). The vesicular exocytosis process is similar between neurons and astrocytes (Crippa et al., 2006), with the principal difference arising in the initiation of exocytosis. Neuronal exocytosis is initiated by the arrival of an action potential at the synapse, which results in membrane depolarization in addition to an influx of Ca^2+^ through transmembrane channels involved in the action potential response; the Ca^2+^ wave initiates a cascade of signaling that results in vesicular release. Astrocytes cannot generate action potentials, but the astrocytic vesicular release also requires a transient increase in intracellular Ca^2+^ concentration. Astrocytes have multiple mechanisms to accomplish this Ca^2+^ increase, including activation of ionotropic or metabotropic receptors and the subsequent inositol 1,4,5-trisphosphate (IP3) signaling cascade, activation of transient receptor potential (TRP) channels, or the release of Ca^2+^ by mitochondria (Guerra-Gomes et al., 2018); or selective activation of either purinergic receptors P2Y1 or protease-activated receptor 1 (PAR-1) that in astrocytes leads to increased intracellular Ca^2+^ concentration (Shigetomi et al., 2008).

Furthermore, recent evidence indicates that astrocytes can release glutamate by the interaction of metabotropic receptors PAR-1 with either the two-pore domain potassium channel (TREK-1) or the Bestrophin-1 (Best-1), a Ca^2+^-activated chloride channel (Figure 2). Glutamate efflux from the intracellular space occurs when the TREK-1 intracellular domain interacts with PAR-1 allowing fast glutamate transient currents, whereas the interaction of PAR-1 and Best-1 leads to slow transient currents (Woo et al., 2012; Lalo et al., 2021). Taken together, the evidence supports the view that both neurons and astrocytes contribute to glutamatergic signaling.

### Glutamate receptors

Glutamatergic receptors are widely distributed in the different regions of the central nervous system. Neurons and astrocytes express glutamate receptors, which split into two families. Ionotropic glutamate receptors (iGluRs; Kukley et al., 2001) and metabotropic glutamate receptors (mGluRs; Schools and Kimelberg, 1999; Fiacco and McCarthy, 2004; Perea and Araque, 2007; Cavaccini et al., 2020).

### iGluRs

The iGluRs family, consisting of NMDA (N-methyl-D-aspartate) receptors, and the AMPA (α-amino-3-hydroxyl-5-methyl-4-isoxazol-propionate) and KA (kainic acid) receptors, which are responsible for the excitatory transmission in the central nervous system of vertebrates. These receptors are ligand-gated ion channels that allow the movement of cations such as Na^+^, K^+^, and Ca^2+^ across the cell membrane. Structurally, iGluRs are transmembrane proteins composed of four subunits that form a central ionic pore comprised of an extracellular amino-terminal domain, an extracellular ligand-binding domain, four transmembrane domains, and an intracellular carboxyl-terminal domain (Traynelis et al., 2010).

### NMDA receptor

NMDA receptors are highly expressed in the brain, and their role in physiological and pathological mechanisms have been studied extensively (Lakhan et al., 2013; Zhou et al., 2013; Intson et al., 2022). The subunits that constitute these receptors are GluN1, GluN2 [A, B, C, D], GluN3A, and GluN3B. A functional receptor contains a tetrameric assembly with two possible configurations. All receptors must have two GluN1 subunits. The remaining two subunits can be a pair of GluN2 subunits, or one GluN2 subunit together with one GluN3 subunit (Schorge et al., 2005; Ulbrich and Isacoff, 2008; Traynelis et al., 2010; Hansen et al., 2021). Moreover, mRNA editing and alternative splicing of the different NMDA subunit genes confer more complex properties to the NMDA receptors’ conformation, which have been extensively reviewed by Hansen and colleagues (Hansen et al., 2021).

Full activation of NMDA receptors in neurons requires membrane depolarization to displace the Mg^2+^ ion that blocks the ion channel with the simultaneous binding of glutamate and the co-agonist, glycine. When both conditions are met, NMDA receptor activation allows Na^+^ and Ca^2+^ ion influx. In addition to glycine, D-serine is an NMDA receptor co-agonist, which is supplied by astrocytes (Henneberger et al., 2010). However, recent evidence indicated that neurons also contribute to the *de novo* synthesis of D-serine (Neame et al., 2019). D-serine metabolism depends on 3-phosphoglycerate dehydrogenase (Phgdh) enzyme activity and glycine concentration (Shibasaki et al., 2017; Neame et al., 2019). Since astrocytes require Phgdh to synthesize L-serine from glucose, astrocyte *de novo* synthesis of L-serine may be a previously unrecognized regulatory mechanism for the NMDA receptors co-agonism by D-serine (Masuoka et al., 2019; Neame et al., 2019).

On the other hand, astrocytes also express NMDA receptors, as evidenced by the presence of GluN1 and GluN2 subunit mRNA (Jimenez-Blasco et al., 2015). In astrocytes, the NMDA receptors are insensitive or weakly sensitive to the blockade of Mg^2+^ ion, and their activation might even occur at negative resting potential (Verkhratsky and Kirchhoff, 2007). Interestingly, the expression pattern of NMDA subunits in astrocytes varies depending on the brain region. For example, cortical astrocytes express GluN1, GluN2A/B, GluN2C, and GluN2D subunits (Conti et al., 1996; Palygin et al., 2011). GluN2B is more abundant in the Bergmann glia cells (Luque and Richards, 1995), whereas the GluN2C is predominantly expressed in the telencephalon glial cells (Alsaad et al., 2019). In hippocampal cultured astrocytes, GluN1 and GluN2 subunits are predominant (Araque et al., 1998a). *In vivo* evaluation of the GluN2C NMDA subunit localization showed that parvalbumin-positive neurons in the globus pallidus, ventral pallidum, and substantia nigra express this subunit, whereas GluN2C in the cortex, striatum, hippocampus, and amygdala colocalizes with astrocytes’ markers (Ravikrishnan et al., 2018). The variety of NMDA subunit compositions confers different activation properties to the receptors, which, in combination with the regional expression patterns, results in differences in the contribution of NMDA receptors to neuronal and astrocyte activity (Palygin et al., 2011).

### AMPA receptor

An AMPA receptor is composed of four subunits from the proteins GluA1, GluA2, GluA3, and GluA4 (Traynelis et al., 2010). AMPA receptors can either be homomers or heteromers. These subunit types are differentially expressed in the nucleus accumbens, dorsal striatum, prefrontal cortex, and hippocampus (Reimers et al., 2011). Activation of AMPA receptors allows Na^+^ and K^+^ influx, but if the receptor conformation lacks a GluA2 subunit or contains a post-transcriptionally modified GluA2 by RNA editing at the Q/R site, the channel will also be Ca^2+^ permeable (Traynelis et al., 2010; Hansen et al., 2021). AMPA subunits interact with the transmembrane AMPA receptor regulatory protein (TARP), which modulates channel opening (Hansen et al., 2021). Activation of AMPA receptors leads to membrane depolarization, leading to the displacement of the Mg^2+^ ion that blocks the NMDA ion channel allowing its activation.

Some studies suggest the presence of AMPA receptors in glial cells (Müller et al., 1992). These receptors have been described in cortical cultured astrocytes (David et al., 1996), and a subpopulation of hippocampal astrocytes express GluA1, GluA2, GluA3, and GluA4 subunits (Matthias et al., 2003). Regarding function, it has been shown that the AMPA receptors modulate the inward-rectifier potassium channels (also known as Kir) in hippocampal astrocytes, which induces gliotransmitter release and AMPA activation in neurons (Schröder et al., 2002; Fiacco and McCarthy, 2004). Of note, AMPA receptors containing at least one GluA3 or GluA4 subunit are permeable to Ca^2+^, as described in hippocampal astrocytes (Seifert and Steinhauser, 1995), providing a direct mechanism to raise intracellular Ca^2+^ concentrations.

### KA receptor

Among the iGluRs family proteins, KA receptors comprise the least studied class (Meyerson et al., 2016). The subunits that form the KA receptors are GluK1, GluK2, GluK3, GluK4, and GluK5. The KA channel is permeable to Na^+^ and K^+^. Some of the most important functions of these receptors are the regulation of synaptic activity (Fernandes et al., 2009) and neuronal plasticity (Lauri et al., 2006).

The expression of receptor subunits does indeed vary across species and brain regions. For example, the primate neocortex primarily expresses the GluK1-2-3 subunits (Huntley et al., 1993), whereas the rodent cortex expresses more GluK2 and GluK4 subunits (Herb et al., 1992). Hippocampal interneurons express KA receptors (Liu et al., 2004). Astrocytes in the hypothalamic arcuate nucleus express GluK1–3 subunits (Diano et al., 1998). Hippocampal astrocytes express GluK2 (Matschke et al., 2015). Some KA subunits allow Ca^2+^ efflux, which in astrocytes can contribute to glutamate vesicular release or activate other Ca^2+^-dependent signaling pathways (see Guerra-Gomes et al., 2018). Interestingly, after chemoconvulsive status epilepticus of temporal lobe epilepsy, CA1 hippocampal reactive astrocytes expressed GluK1, GluK2/3, GluK4, and GluK5 (Vargas et al., 2013). Of these, GluK1 and GluK5 expression in astrocytes persist during the presence of spontaneous seizures, suggesting that KA receptors in astrocytes might contribute to the pathophysiology of epilepsy (Vargas et al., 2013).

In general, iGluRs are a key component for synaptic activity and neuronal processing, however, more research is required to elucidate the contribution of each iGluRs in astrocytes and how it contributes to glutamatergic neurotransmission.

### mGluRs

mGluRs are coupled to G-proteins and modulate slow synaptic transmission through second messengers. To date, eight mGluRs (mGluR1–8) have been described and divided into three groups designated I, II, and III according to similarities in their distinctive features: gene sequence, pharmacological properties, and intracellular signaling mechanisms (Sladeczek et al., 1985).

The group I receptors, mGluR1 and mGluR5, are associated with intracellular Ca^2+^ signaling, phospholipase C, and these receptors are mainly activated by 3,5-dihydroxyphenylglycine (DHPG). Group II includes mGluR2 and mGluR3, which are negatively coupled to adenylate cyclase and are selectively activated by LY379268. Finally, group III contains mGluR4, mGluR6, mGluR7, and mGluR8 receptors, which, like group II, are negatively coupled to the adenylate cyclase (Sugiyama et al., 1987; Masu et al., 1991).

Receptors from the Group I mGluRs are more widespread in the brain. They are expressed in neurons from the olfactory bulb, cerebral cortex, globus pallidus, lateral septum, cerebellar Purkinje cells, and thalamic nuclei (Crupi et al., 2019). Group II mGluRs are expressed in the olfactory bulb and cerebellar cortex (for more details see Crupi et al., 2019). In contrast, astrocytes showed a predominance of mGluR1, mGluR3, and mGluR5 receptors, which have been described thus far in the hippocampus and cerebral cortex (Schools and Kimelberg, 1999; Sun et al., 2013; Spampinato et al., 2018). Using electron microscopy and immunohistochemistry, mGluR2 and mGluR3 have been identified in astrocytes in the rat ventrobasal thalamus (Mineff and Valtschanoff, 1999).

The activation of mGluR3 and mGluR5 increases Ca^2+^ intracellular concentration, triggering vesicular glutamate release in neurons and astrocytes, which influences synaptic activity and plasticity (Fiacco and McCarthy, 2004; Perea and Araque, 2007; Cavaccini et al., 2020). Specific activation of metabotropic group II receptors in astrocyte cultures increases the expression of GLAST (Gegelashvili et al., 2000). Likewise, activating the mGluR5 receptor increases glutamate uptake through increased expression of GLT-1 and GLAST transporters (Vermeiren et al., 2005). However, contradicting results have been observed during the activation of group I mGluRs, which reduces the expression of GLAST (Gegelashvili et al., 2000). Therefore, more studies are needed to understand the specific role of each mGluR in astrocytic glutamate transporter expression and function.

#### Glutamate transporters

Glutamate transporters, also known as excitatory amino acid transporters (EAATs), maintain optimal extracellular glutamate concentration; these transporters belong to the solute carrier (SLC) family 1 (high-affinity glutamate transporters; He et al., 2009). These proteins are expressed in neurons and glial cells, especially astrocytes, and are responsible for the bulk of glutamate uptake (Rothstein et al., 1996) by the co-transport of glutamate, Na^+^ (three molecules), H^+^ (one molecule), and counter-transport of K^+^ (one molecule; Levy et al., 1998).

Five EAATs have been identified in humans. In rodents, these transporters were named excitatory amino acid carrier 1 EAAC1/EAAT3, GLAST/EAAT1, GLT-1/EAAT2, excitatory amino acid transporter 4 (EAAT4), and excitatory amino acid transporter 5 (EAAT5; Figure 2; Kanai and Hediger, 1992; Pines et al., 1992; Storck et al., 1992; Fairman et al., 1995; Rothstein et al., 1996; Arriza et al., 1997).

Neurons in the rat cerebral cortex, hippocampus, cerebellum, and spinal cord express the EAAC1 transporters (Kanai et al., 1995; Shashidharan et al., 1997). Interestingly, this transporter is mainly involved in anion conductance and uptake of cysteine, a precursor of glutathione synthesis (Lee et al., 2020). EAAT4 is highly expressed in cerebellar Purkinje cells (Magi et al., 2019). However, it is also found in the fore- and mid-brain and the somatosensory cortex (Massie et al., 2008; de Vivo et al., 2010). EAAT5 is mainly expressed in the retina (Arriza et al., 1997).

GLAST and GLT-1 are highly expressed in astrocytes of the hippocampus, striatum, and cerebral cortex and oversee glutamate uptake at the synapse (Levy et al., 1993; Lehre et al., 1995; Bergles and Jahr, 1997; Mennerick et al., 1998). According to studies focused on evaluating the subcellular distribution of GLAST and GLT-1, both transporters are highly expressed in hippocampal astrocytes with a predominant presence of GLT-1 in the filopodium and perivascular end-feet, and GLAST is mostly present in the soma and processes (Schreiner et al., 2014; Radulescu et al., 2022). However, the GLT-1 isoforms (GLT-1a and b) are expressed in neurons from the hippocampus, cerebral cortex, striatum, thalamus, and midbrain (Chen et al., 2002, 2004; Berger et al., 2005). Astrocyte processes express more GLT-1a mRNA, whereas GLT-1b mRNA has been detected mainly in the cell body (Berger et al., 2005). In neurons, GLT-1a protein expression in axons, spines, and dendrites contributes to glutamate reuptake in the excitatory terminals (Chen et al., 2004). It has been suggested that GLT-1 in neurons provides glutamate as a substrate for energy metabolism and mitochondrial functionality (Petr et al., 2015; McNair et al., 2019).

Astrocytes are the main regulators of extracellular glutamate concentration through the GLT-1 and GLAST glutamate transporters; expression of these transporters is regulated by neuronal activity (Swanson et al., 1997; Perego et al., 2000). Interestingly, besides neurons, brain endothelial cells can also induce GLT-1 expression through Notch signaling (Lee et al., 2017).

In addition to EAATs, the SLC7A11/xCT transporter is a cystine/glutamate antiporter, which transports a cystine into the cell while exchanging for glutamate (1–1 ratio), in a sodium-independent fashion; therefore, contributing to astrocyte glutamate release (Bannai, 1986). It consists of two subunits, the light subunit (SLC7A11) and the heavy subunit (SLC3A2). Whereas the light subunit is responsible for the active transport of cystine and glutamate, the heavy subunit is necessary for intracellular trafficking and proper membrane arrangement of the transporter (Nakamura et al., 1999; Shin et al., 2017). The SLC7A11/xCT transporter takes up cystine and, inside the cell, cystine will be converted to cysteine, the main precursor for the antioxidant glutathione (Conrad and Sato, 2012). SLC7A11/xCT is highly expressed in the human brain (Sato et al., 1999). In mice, SLC7A11/xCT is prominently expressed in the hippocampus, cortex, hypothalamus, and dentate gyrus (Sato et al., 2002). The SLC7A11/xCT expression occurs mainly in glial cells (Re et al., 2006), including astrocytes (Ottestad-Hansen et al., 2018). SLC7A11/xCT is essential to avoid oxidative damage (Lewerenz et al., 2012), probably due to its link with glutathione synthesis. It follows that blocking SLC7A11/xCT leads to an increase in oxidative stress and astrocyte death (Chen et al., 2000), a process known as oxidative glutamate toxicity (Schubert and Piasecki, 2001).

Interestingly, *Drosophila* xCT gene-knockout reduced the extracellular ambient glutamate concentration by 50%, suggesting that the xCT transporter is essential for extracellular glutamate regulation (Augustin et al., 2007). Also, the use of sulfasalazine, an xCT inhibitor, reduces the NMDA-induced current by 66.8% in mouse hippocampus slices, indicating that glutamate release through xCT contributes to neuronal activation (Koh et al., 2022). In addition, xCT deletion in mice induces an age-dependent anxiety-like behavior (Bentea et al., 2015). In a related experiment, exposing the astrocytoma-derived cell line (1321N1) to peroxide increased both ambient glutamate concentrations and the population of xCT transporters, suggesting that xCT activity contributes to glutamate release and accumulation (Kazama et al., 2020). Further experiments are necessary to clarify whether the release of glutamate by the xCT transporter contributes to the activation of iGluRs and mGluRs in neurons or astrocytes and if it contributes to pathological processes *in vivo*.

## Role of astrocytes in glutamatergic neurotransmission during physiological conditions

Astrocyte function was initially thought to support neuronal activity or protect neurons from excitotoxicity. However, later studies suggested that astrocytes can directly or indirectly modulate synaptic neuronal activity (Figure 2; Nedergaard, 1994; Beppu et al., 2021) and influence behavior (Lyon and Allen, 2022). The first level of regulation is glutamate uptake by astrocytic transporters as they regulate the neurotransmitter levels in the synaptic cleft; these transporters indirectly modulate neuronal transmission (Jabaudon et al., 1999), neuronal activity (Estrada-Sánchez et al., 2019) and survival (Estrada-Sánchez et al., 2007, 2019). Glutamate uptake can also regulate the availability of glutamine to synthesize glutamate. As mentioned in an earlier section, once astrocytes take up glutamate, it can be metabolized into α-ketoglutarate by glutamate dehydrogenase or into glutamine through amidation of glutamate by the glutamine synthetase (as part of the glutamate/glutamine cycle; Laake et al., 1999; Islam et al., 2010). As this enzyme is highly expressed in astrocytes, they are considered the major glutamine reservoir and an important source of precursor for the metabolism of glutamate and gamma-aminobutyric acid (GABA; Hamberger et al., 1979; Norenberg and Martinez-Hernandez, 1979). Therefore, astrocytes also might regulate glutamatergic neuronal dynamics by the amount of glutamine released into the synaptic cleft.

Astrocytes can also regulate neuronal activity by releasing gliotransmitters such as glutamate, ATP, D-serine, or GABA, also known as gliotransmission. Once the gliotransmitter is released, it activates its target receptors and, consequently, generates responses in the same astrocyte (autocrine response) or nearby cells, including neurons (Lapato and Tiwari-Woodruff, 2018; Savtchouk and Volterra, 2018; Beppu et al., 2021; Sherwood et al., 2021a). In addition to gliotransmission, astrocytes contribute to neuronal activity by regulating the availability of NMDA receptor co-agonists (glycine and D-serine; Sherwood et al., 2021a). However, more studies are needed to better understand this process’s physiological and pathological implications. For a more detailed description of this topic, see Sherwood et al. (2021a). Also, astrocytes can release active molecules through hemichannels (Lee et al., 2011; Montero and Orellana, 2015; Lalo et al., 2021). This topic is beyond the scope of this review, but for more information refer to Sahlender et al. (2014), Montero and Orellana (2015), and Caudal et al. (2020).

Experiments using electrophysiology, optical imaging, and molecular biology demonstrated that astrocytes respond to neurotransmitters. Activation of mGluR initiates a cellular signaling cascade that increases intracellular Ca^2+^ concentration on a timescale of about 50–200 ms (Batchelor and Garthwaite, 1997; Marcaggi et al., 2009), in contrast to the comparatively fast ionotropic receptors that take approximately 1–10 ms in neurons to initiate the same response (Traynelis et al., 2010; Reiner and Levitz, 2018). iGluRs are fast-acting because extracellular Ca^2+^ directly enters the cell through the open channel, although a significant Ca^2+^ concentration rise requires a substantial number of simultaneously open iGluR channels. The activation of mGluR initiates a cellular signaling cascade that amplifies the input signal, albeit at the cost of response time; the activated mGluR activates the phospholipase C/IP3 pathway, which then generates the release of Ca^2+^ from the endoplasmic reticulum (Decrock et al., 2013; Rodriguez-Prados et al., 2020). This intracellular source of Ca^2+^ induces gliotransmitter release by Ca^2+^-dependent exocytosis (Bezzi et al., 1998, 2004; Zhang et al., 2004; Mothet et al., 2005; Crippa et al., 2006; Woo et al., 2012; Li et al., 2013b; Navarrete et al., 2013; Heller et al., 2020; Takata-Tsuji et al., 2021). Gliotransmitter release in turn affects neuronal functioning, forming a feedback loop.

Although astrocytes express both iGluRs and mGluRs, intracellular Ca^2+^ concentration rises mainly due to the activation of mGluRs rather than iGluRs (Conti et al., 1996; Schools and Kimelberg, 1999). However, we cannot dismiss the contribution of iGluRs in astrocytes for two reasons. First, the temporal dynamics of iGluRs are approximately an order of magnitude faster than mGluRs. Second, the presence of certain subunits confers different Ca^2+^ permeability to iGluRs (Seifert et al., 1997; Brand-Schieber and Werner, 2003; Brand-Schieber et al., 2004; Palygin et al., 2010). The subunits that increase Ca^2+^ permeability are GluN3 subunits for NMDA (Cull-Candy et al., 2001; Kvist et al., 2013), GluA2 for AMPA (Traynelis et al., 2010), and GluK3-4 for KA (Burnashev et al., 1995). The ratio of iGluRs with and without increased Ca^2+^ permeability in astrocytes may constitute a regulatory mechanism to modulate Ca^2+^ influx into the cell, affecting Ca^2+^-dependent pathways.

The release of glutamate from astrocytes contributes to NMDA-related long-term depression. This mechanism is initiated by cannabinoid receptor type 1 (CB1) activation of astrocytes, which increases Ca^2+^ concentration and produces astrocytic glutamate release. Increased extracellular glutamate activates NMDA receptors, promoting internalization of AMPA receptors (Han et al., 2012; Min and Nevian, 2012) in the cortex and hippocampus. In the cortex, the activation of astrocytic CB1 receptors by exogenous cannabinoids impairs spatial working memory (Han et al., 2012; Min and Nevian, 2012).

In the striatum, two subpopulations of astrocytes may selectively regulate the response for dopamine D1 vs. D2 medium spiny neuron (MSN) subpopulations. Astrocytic CB1 activation elicits astrocytic glutamate release that specifically induces activity from either D1 or D2 MSNs, but not both (Martín et al., 2015). This finding suggests nuanced organization and interaction of neurons and astrocytes in the striatum.

On the other hand, Beppu et al. (2021) demonstrated that Bergmann glial cells amplify excitatory neuronal signals in the cerebellar cortex by releasing glutamate through a mechanism involving bicarbonate efflux and resultant intracellular acidification, a mechanism sensitive to the inhibition of volume-regulated ion channels. Although this is a novel and exciting mechanism by which astrocytes can regulate the neuronal activity, more experiments are needed to elucidate its specific molecular components and their contribution during physiological conditions *in vivo*. Also, this opens the question of whether this is a mechanism restricted to the cerebellar cortex or if it also occurs in other brain areas.

Interneurons also play an important role in astrocyte modulation of neuronal activity. Interneurons are locally projecting neurons that regulate neuronal activity levels through inhibitory signaling that counteracts excitatory (e.g., glutamatergic) signaling, and helps to prevent runaway excitatory cascades. For example, in the hippocampus, stimulation of inhibitory GABAergic interneurons activates GABA_B_ receptors in astrocytes, which subsequently triggers increased Ca^2+^ waves in the surrounding astrocytes, potentiating pyramidal inhibition. This effect is blocked by glutamatergic antagonist CNQX [cyanquixaline (6-cyano-7-nitroquinoxaline-2,3-dione)] and AP5 (2-amino-5-phosphopentanoic acid), suggesting that interneuron-astrocyte-mediated potentiated inhibition of pyramidal neurons depends on astrocyte-mediated glutamate release (Kang et al., 1998). Also, in the hippocampus, Perea et al. (2016) described that, besides activating GABA_B_ receptors in astrocytes, GABA release by interneurons also activates GABA_A_ receptors in presynaptic neurons, which inhibited synaptic activity. However, when the interneuron leads to astrocyte-mediated glutamate release, presynaptic activation of mGluR 1/5 receptors contributes to synaptic potentiation (Kang et al., 1998; Perea et al., 2016). In the mouse cerebellar cortex, activation of purinergic P2Y1 receptors and AMPA receptors in Bergmann glial cells leads to glutamate-vesicular release that activates NMDA receptors in the interneurons, enhancing the inhibitory synaptic input to Purkinje cells (Rudolph et al., 2016).

Shen et al. (2017) described that autocrine activation of P2Y1 purinergic receptors in astrocytes modulates the release of glutamate mediated by the Ca^2+^-dependent chloride channel Best-1 and the subsequent activation of extra-synaptic NMDA receptors in neurons. Related studies evaluated the effect of astrocytic Ca^2+^-dependent glutamate release on the activity of neuronal extra-synaptic NMDA receptors (Le Meur et al., 2007; Shen et al., 2017; Koh et al., 2021). These receptors contain the GluN2B subunit and their activation produce a slow inward current with an amplitude of 18–477 pA, with a rise time of 13–332 ms, and decay times of 72–1,630 ms. The presence of extra-synaptic NMDA currents directly depends on astrocytes’ intracellular Ca^2+^ concentrations; they decrease when Ca^2+^ signaling is abolished and increase when intracellular Ca^2+^ concentration rises (Araque et al., 1998b; Pirttimaki et al., 2011; Perea et al., 2014); these neuronal currents are generated by astrocyte activity in the hippocampus, cortex (Gomez-Gonzalo et al., 2017, 2018), and nucleus accumbens (Corkrum et al., 2019).

Moreover, Santello et al. (2011) showed that the P2Y1 activation and consequent Ca^2+^-dependent glutamate release in astrocytes from the dentate gyrus also involve the tumor necrosis factor-alpha (TNFα), which at physiological concentrations (pM range), favors the adequate exocytosis of astrocytic glutamatergic vesicles. Interestingly, glutamate released activates presynaptic NMDA receptors, particularly expressing the GluN3A subunit with a low voltage-dependent Mg^2+^ block (Savtchouk et al., 2019). In an excellent review, Di Castro and Volterra (2022) describe how this mechanism might be relevant within the entorhinal cortex-dentate gyrus circuit involved in memory processing.

Additional evidence beyond the hippocampus indicates that astrocytes have a functional role in behavior regulation. For example, astrocytes in the suprachiasmatic nucleus increase Ca^2+^ signaling at night. This signaling is highly related to circadian behavior regulated by glutamate release (Brancaccio et al., 2019). Blum et al. (2021) demonstrated in *Drosophila melanogaster* through *in vivo* two-photon experiments that increased astrocyte Ca^2+^ activity correlates with sleep needs. Another behavior modulated by astrocytes is feeding (Sweeney et al., 2016; Varela et al., 2021). Specific stimulation of astrocytes from mice’s medial basal hypothalamus suppresses food intake (Sweeney et al., 2016). Finally, mice lacking astrocyte glucocorticoid receptors in the amygdala show attenuated anxiety behaviors in the open field behavioral test and fear memory (Wiktorowska et al., 2021), demonstrating a direct involvement of astrocytes in fear memory and anxiety.

The extensive evidence reflects the key role of Ca^2+^-mediated signaling in astrocytes, which requires stimulation of IP3 receptors (IP_3_R). There are three subtypes of IP_3_R in mammals (IP_3_R1, IP_3_R2, and IP_3_R3) and among them, IP_3_R2 was widely accepted as the only functional subtype in astrocytes (Sherwood et al., 2021b). However, recent evidence showed that IP_3_R1 and IP_3_R3 are also present in astrocytes and participate in Ca^2+^-mediated signaling, especially in astrocytic processes (Sherwood et al., 2017, 2021b). This new finding raises questions about the functional role and subcellular localization of the different IP_3_R subtypes in astrocytes during gliotransmission and its relevance during physiological processes.

As a whole, this evidence indicates that gliotransmission modulates the activity of astrocytes and neuronal circuits (projecting and interneurons) and appears to be a widespread mechanism in the brain. Besides glutamate, ATP purinergic receptors emerge as a key component that triggers astrocytes’ modulation of neuronal circuits. Although the current evidence indicates that gliotransmission can influence behavior, more research is needed to dissect the different roles of astrocytes in shaping animal behavior.

## Role of astrocytes in glutamatergic neurotransmission during pathological conditions

Alterations in the glutamate transporters are related to neurodegenerative diseases (Pajarillo et al., 2019). *Postmortem* brains of Alzheimer’s disease patients showed a reduction in EAAT1 and EAAT2 (Masliah et al., 2000). Epilepsy is associated with decreased EAAT2 (Tanaka et al., 1997). In the intrahippocampal kainic acid model of temporal lobe epilepsy, GLT-1 and GLAST expression increase early after the treatment, suggesting that dysregulation in the expression of astrocyte glutamate transporters could contribute to the development of epilepsy. However, the accuracy of this hypothesis has not yet been determined (Peterson and Binder, 2019). In Huntington’s disease, an inherited neurodegenerative disorder, reduced expression of EAAT2 was observed in *postmortem* brain samples (Faideau et al., 2010). In the R6/2 Huntington’s disease transgenic mouse model, decreased content of GLT-1 and GLAST correlates with increased vulnerability to glutamate-induced toxicity (Estrada-Sánchez et al., 2009, 2010). Likewise, cortical pyramidal neurons in the R6/2 mice are more vulnerable to glutamate-mediated paroxysmal activity during the inhibition of both GLT-1 and GLAST transporters (Estrada-Sánchez et al., 2019).

To date, exploration of how gliotransmission contributes to neural information processing and behavior focused mostly on physiological conditions. Less is known about how changes in gliotransmission contribute to the pathology of neurological, neuropsychiatric, or neurodegenerative conditions. However, data from physiological studies point out that astrocytes in the striatum can modulate differentially D1 or D2 MSNs (see above), and its dysfunction may contribute to diseases like Parkinson’s and Huntington’s disease. D1 and D2 MSNs comprise key components of the brain circuits that control movement and, at the behavioral level, both diseases involve substantial alterations in movement control. At the cellular level, D1/D2 MSN impairment contributes to neuropathology, and a distinct line of evidence demonstrates altered astrocyte functioning (for a review, see Estrada-Sánchez et al., 2017). The results from Martín et al. (2015) suggest that these two lines of evidence from disease pathology are connected to healthy tissue function (Martín et al., 2015); further understanding of how this system works may yield promising pathways for future therapeutic interventions for these diseases.

Another possible contribution of gliotransmission to neuropathology might be through the activation of extra-synaptic NMDA receptors since the NMDA-mediated response in neurons depends on its subcellular localization. Whereas activation of synaptic NMDA receptors leads to survival pathways, activation of extra-synaptic NMDA receptors initiates neuronal death cascades (Kaufman et al., 2012). In fact, it has been documented that activation of extra-synaptic NMDA receptors contributes to the neurodegenerative processes described in Huntington’s disease and ischemia (Hardingham and Bading, 2010; Milnerwood et al., 2012). Because activation of extra-synaptic NMDA receptors indicates astrocytic Ca^2+^-dependent glutamate release, it is important to evaluate the possible role of astrocytes in the balance/imbalance between synaptic and extra-synaptic glutamatergic receptors activation and whether this contributes to survival or neuronal death pathways.

The description of three functional IP_3_R subtypes in astrocytes raises new questions about their role in pathological states. Recently, it was shown that the IP_3_R1 subtype has a key role in chronic itching (Shiratori-Hayashi et al., 2021) and the absence of IP_3_R2 in astrocytes generates autism spectrum disorder-like behaviors (Wang et al., 2021).

More studies are needed to fill the knowledge gaps about the contribution of gliotransmission during the pathological process of neurodegenerative, psychiatric, and neuropathological conditions.

## Perspectives

The evidence reviewed strongly suggests a complex functional interaction between neurons and astrocytes. The extent to which astrocytes modulate the synapse could vary depending on the brain region, influencing its information processing and behavioral output. To date, most of the information on gliotransmission has been centered in the hippocampus, and less is known about gliotransmission in other areas of the brain. Similarly, most studies have focused on physiological conditions, and although there is evidence that astrocytes contribute to the neuropathological process, the precise role of astrocytes during neuropathological processing is still to be determined. The review also suggests extensive opportunities for further research, including the specific contribution of each gliotransmitter described to date and perhaps the identification of new gliotransmitters, their synthesis, and release systems. It is also important to better understand the effects of gliotransmitters on neighboring cells, including the same astrocyte or afferent, efferent neurons, interneurons, and microglia cells. Finally, additional studies will clarify the functional interconnection among different signaling pathways in a tripartite synapse, such as glutamatergic, purinergic, and GABAergic, during physiological and pathological conditions. Current evidence is limited to *in vitro* and brain slice experiments, which limits our understanding of the functional role of all these components *in vivo*.

## Conclusions

The development of new experimental tools has widened our understanding of the synapse, where astrocytes emerge as a complex contributor. The evidence indicates that astrocyte release of gliotransmitters such as glutamate, ATP, D-serine, and GABA can activate, potentiate, or inhibit the activity of projection neurons, interneurons, or other astrocytes. Furthermore, astrocytes can influence neuronal synaptic modification through effects on long-term depression and potentiation. It is likely that more than one gliotransmitter coexists within the same astrocyte, providing a new degree of complexity to astrocyte modulatory activity. These mechanisms suggest additional layers of information-processing capability, enabled by astrocytes that extend beyond the traditional focus on neurons as the information-processing cells of the brain. More studies are needed to understand how astrocytes modulate neuron-astrocyte network activity across the brain during physiological and pathological conditions. Finally, deepening the understanding of the functional dynamics between gliotransmitter signaling in neurons and astrocytes will widen the therapeutic targets for neuropathological conditions and neurodegenerative diseases, including ischemia, stroke, and Huntington’s disease.

## Author contributions

AOC-S and AME-S conceived the review topic. AOC-S, VMR-R, TBM-B, AP-S, AGH, DPP-P, AMG, and AME-S prepared the first draft and figures. All authors contributed to the article and approved the submitted version.
